# Supplementary material for: Structure determination of ζ-N2 from single-crystal X-ray diffraction and theoretical suggestion for the formation of amorphous nitrogen
Source: Nat Commun. 2023 Oct 5;14:6207. doi: 10.1038/s41467-023-41968-2 (PMC10556017; doi:10.1038/s41467-023-41968-2)
Supplement: Supplementary file 1 — Supplementary Information [file 41467_2023_41968_MOESM1_ESM.pdf]

Supplementary Information

Structure determination of ζ-N_2 from single-crystal X-ray diffraction and theoretical suggestion for the formation of amorphous nitrogen

Dominique Laniel,[1*] Florian Trybel,[2*] Andrey Aslandukov,[3,4] James Spender,[1] Umbertoluca Ranieri,[1] Timofey Fedotenko,[5] Konstantin Glazyrin,[5] Eleanor Lawrence Bright,[6] Stella Chariton,[7] Vitali B. Prakapenka,[7] Igor A. Abrikosov,[2] Leonid Dubrovinsky[4] and Natalia Dubrovinskaia[2,3]

Affiliations:

[1]Centre for Science at Extreme Conditions and School of Physics and Astronomy, University of Edinburgh, EH9 3FD Edinburgh, United Kingdom

[2]Department of Physics, Chemistry and Biology (IFM), Linköping University, SE-581 83, Linköping, Sweden

[3]Material Physics and Technology at Extreme Conditions, Laboratory of Crystallography, University of Bayreuth, 95440 Bayreuth, Germany

[4]Bayerisches Geoinstitut, University of Bayreuth, 95440 Bayreuth, Germany

[5]Photon Science, Deutsches Elektronen-Synchrotron, Notkestrasse 85, 22607 Hamburg, Germany

[6]The European Synchrotron Radiation Facility, 38043 Grenoble Cedex 9, France

[7]Center for Advanced Radiation Sources, The University of Chicago, Chicago, Illinois 60637, United States

*Correspondence to: dominique.laniel@ed.ac.uk and florian.trybel@liu.se

Supplementary Table 1: Crystallographic data for ε-N_2 at 54 GPa. The atomic positions are compared to those found for ε-N_2 at 7.8 GPa and 110 K by Mills *et al*[1]. The crystallographic data has been submitted under the deposition number CCDC 2280044.

	**ε-N_2**
	**Exp.**
Pressure (GPa)	54
Space group, #	*R*-3*c*, 167
Z	24
a (Å)	6.819(8)
b (Å)	6.819(8)
c (Å)	9.749(9)
V (Å^3)	392.6(7)
Refinement details	
Wavelength (λ, Å)	0.2950
μ (mm^{-1})	0.061
# measured/independent reflections ($I \geq 3\sigma$)	247 / 79 (55)
$(\sin \theta/\lambda)_{max}$ (Å^{-1})	0.757
R_{int} (%)	9.08
R_1 (%)	8.86
wR_2 (%)	7.54
R_1 (all data, %)	13.88
wR_2 (all data, %)	8.03
Goodness of fit	3.68
No. of parameters	7
$\Delta\rho_{min}$, $\Delta\rho_{max}$ (eÅ^{-3})	-0.47, 0.66

**Atomic positions**				
Atom	Wyckoff position	Fractional atomic coordinates (x; y; z)		U_{iso} (Å^2)
		**Exp.**	**Literature (7.8 GPa, 110 K)**[1]	
N1	12*c*	0; 0; 0.0529(6)	0; 0; 0.0495	0.022
N2	36*f*	0.0653(6); 0.2696(3); 0.2111(3)	0.0604; 0.2731; 0.2172	0.022

Supplementary Table 2: Crystallographic data for ζ-N_2 at 63 GPa. Some parameters have both the experimental and the calculated value. The crystallographic data has been submitted under the deposition number CCDC 2237807.

	**ζ-N_2**	
	**Exp.**	**Calc.**
Pressure (GPa)	63	63
Space group, #	*C*2/*c*, 15	*C*2/*c*, 15
Z	16	16
a (Å)	7.580(5)	7.626
b (Å)	6.635(6)	6.470
c (Å)	5.018(2)	5.000
β (°)	97.64(4)	95.32
V (Å^3)	250.2(3)	245.6

Refinement details	
Wavelength (λ, Å)	0.2890
μ (mm^{-1})	0.064
# measured/independent reflections ($I \geq 3\sigma$)	261 / 142 (92)
$(\sin \theta/\lambda)_{max}$ (Å^{-1})	0.623
R_{int} (%)	5.26
R_1 (%)	7.57
wR_2 (%)	6.61
R_1 (all data, %)	9.93
wR_2 (all data, %)	7.33
Goodness of fit	1.58
No. of parameters	17
$\Delta\rho_{min}$, $\Delta\rho_{max}$ (eÅ^{-3})	-0.29, 0.29

**Atomic positions**				
Atom	Wyckoff position	Fractional atomic coordinates (x; y; z)		U_{iso} (Å^2)
		**Exp.**	**Calc.**	
N1	8*f*	0.1897(7); 0.2506(8); 0.0337(9)	0.1789; 0.2528; 0.0114	0.0248(16)
N2	8*f*	0.4006(8); 0.1772(8); 0.4431(9)	0.3929; 0.1804; 0.4496	0.0227(14)
N3	8*f*	0.0695(6); 0.0157(8); 0.2755(8)	0.0725; 0.0173; 0.2682	0.0198(13)
N4	8*f*	0.1416(7); 0.4158(8); 0.4039(10)	0.1372; 0.4174; 0.3806	0.0226(13)

Supplementary Table 3: Crystallographic data for ζ-N_2 at 70 GPa. Some parameters have both the experimental and the calculated value. The crystallographic data has been submitted under the deposition number CCDC 2237808.

		**ζ-N_2**		
		**Exp.**		**Calc.**
Pressure (GPa)		70		70
Space group, #		*C*2/*c*, 15		*C*2/*c*, 15
Z		16		16
a (Å)		7.613(5)		7.541
b (Å)		6.577(6)		6.393
c (Å)		4.952(2)		4.943
β (°)		96.73(4)		95.37
V (Å^3)		246.2(3)		237.3
Refinement details				
Wavelength (λ, Å)		0.291		
μ (mm^{-1})		0.065		
# measured/independent reflections ($I \geq 3\sigma$)		166 / 109 (42)		
$(\sin \theta/\lambda)_{max}$ (Å^{-1})		0.595		
R_{int} (%)		7.18		
R_1 (%)		8.38		
wR_2 (%)		8.04		
R_1 (all data, %)		18.66		
wR_2 (all data, %)		10.61		
Goodness of fit		1.20		
No. of parameters		17		
$\Delta\rho_{min}$, $\Delta\rho_{max}$ (eÅ^{-3})		-0.26, 0.37		
**Atomic positions**				
Atom	Wyckoff position	Fractional atomic coordinates (x; y; z)		U_{iso} (Å^2)
		**Exp.**	**Calc.**	
N1	8*f*	0.188(2); 0.2542(14); 0.0300(19)	0.179; 0.2527; 0.0118	0.032(3)
N2	8*f*	0.403(2); 0.1790(15); 0.4430(14)	0.393; 0.1805; 0.4494	0.032(2)
N3	8*f*	0.0735(18); 0.0180(13); 0.2736(15)	0.0727; 0.0173; 0.2682	0.024(2)
N4	8*f*	0.1384(19); 0.4143(13); 0.3990(14)	0.1375; 0.4173; 0.3803	0.030(2)

Supplementary Table 4: Crystallographic data for ζ-N_2 at 86 GPa. Some parameters have both the experimental and the calculated value. The crystallographic data has been submitted under the deposition number CCDC 2261391.

	**ζ-N_2**	
	**Exp.**	**Calc.**
Pressure (GPa)	86	86
Space group, #	*C*2/*c*, 15	*C*2/*c*, 15
Z	16	16
a (Å)	7.417(5)	7.400
b (Å)	6.354(6)	6.232
c (Å)	4.869(2)	4.844
β (°)	96.57(4)	95.51
V (Å^3)	228.0(3)	222.3
Refinement details		
Wavelength (λ, Å)	0.2846	
μ (mm^{-1})	0.070	
# measured/independent reflections ($I \geq 3\sigma$)	226 / 115 (77)	
$(\sin \theta/\lambda)_{max}$ (Å^{-1})	0.876	
R_{int} (%)	2.36	
R_1 (%)	6.16	
wR_2 (%)	5.53	
R_1 (all data, %)	9.44	
wR_2 (all data, %)	5.79	
Goodness of fit	2.49	
No. of parameters	17	
$\Delta\rho_{min}$, $\Delta\rho_{max}$ (eÅ^{-3})	-0.22, 0.23	

**Atomic positions**				
Atom	Wyckoff position	Fractional atomic coordinates (x; y; z)		U_{iso} (Å^2)
		**Exp.**	**Calc.**	
N1	8*f*	0.1816(5); 0.2513(10); 0.0197(11)	0.1768 0.2526 0.0122	0.0220(12)
N2	8*f*	0.3965(6); 0.1784(10); 0.4439(11)	0.3940 0.1821 0.4491	0.0204(13)
N3	8*f*	0.0710(6); 0.0222(14); 0.2747(9)	0.0748 0.0175 0.2684	0.0199(13)
N4	8*f*	0.1421(6); 0.4128(11); 0.3907(11)	0.1380 0.4184 0.3757	0.0197(13)

Supplementary Table 5: Calculated Raman and infrared modes of ζ-N_2 at 70 GPa.

Mode #	Frequency (cm^{-1})	Infrared intensity	Raman intensity
1	156.44739	0.3004	0
2	207.09612	0.1203	0
3	207.40302	0	210.6105
4	217.31589	0	102.5346
5	258.93153	0	80.2641
6	264.96723	0.1762	0
7	273.18192	< 0.00005	0
8	273.81618	0.184	0
9	279.09486	0	149.2734
10	300.10728	0.0544	0
11	302.27604	0	61.4838
12	322.90995	0	29.7639
13	329.88681	0	3.4866
14	335.45193	0	92.394
15	340.46463	0.0863	0
16	345.39549	0.0766	0
17	359.21622	0	19.4373
18	370.17255	0	205.3557
19	377.53815	0.0044	0
20	404.20776	0.0029	0
21	404.33052	0	6.0105
22	408.18723	0	335.5497
23	412.12578	0.1159	0
24	432.88245	0.4258	0
25	444.81063	0	98.8245
26	447.96147	0	826.737
27	463.92027	0.0101	0
28	476.04282	0	54.2955
29	490.57965	0.029	0
30	502.36461	0	583.6251
31	518.93721	0.0571	0
32	530.56872	0	0.5979
33	538.61973	0.0011	0
34	565.50417	0.0127	0
35	577.93362	0.7998	0
36	580.041	0	365.3568
37	594.79266	0	136.6827
38	2389.95306	0	31664.2584
39	2409.95271	0.0001	0
40	2429.37948	0	33.4566
41	2442.77055	0	4569.456
42	2453.79849	0.2867	0
43	2454.90333	0.0146	0
44	2461.53237	0	362.5341
45	2471.83398	0	844.8627

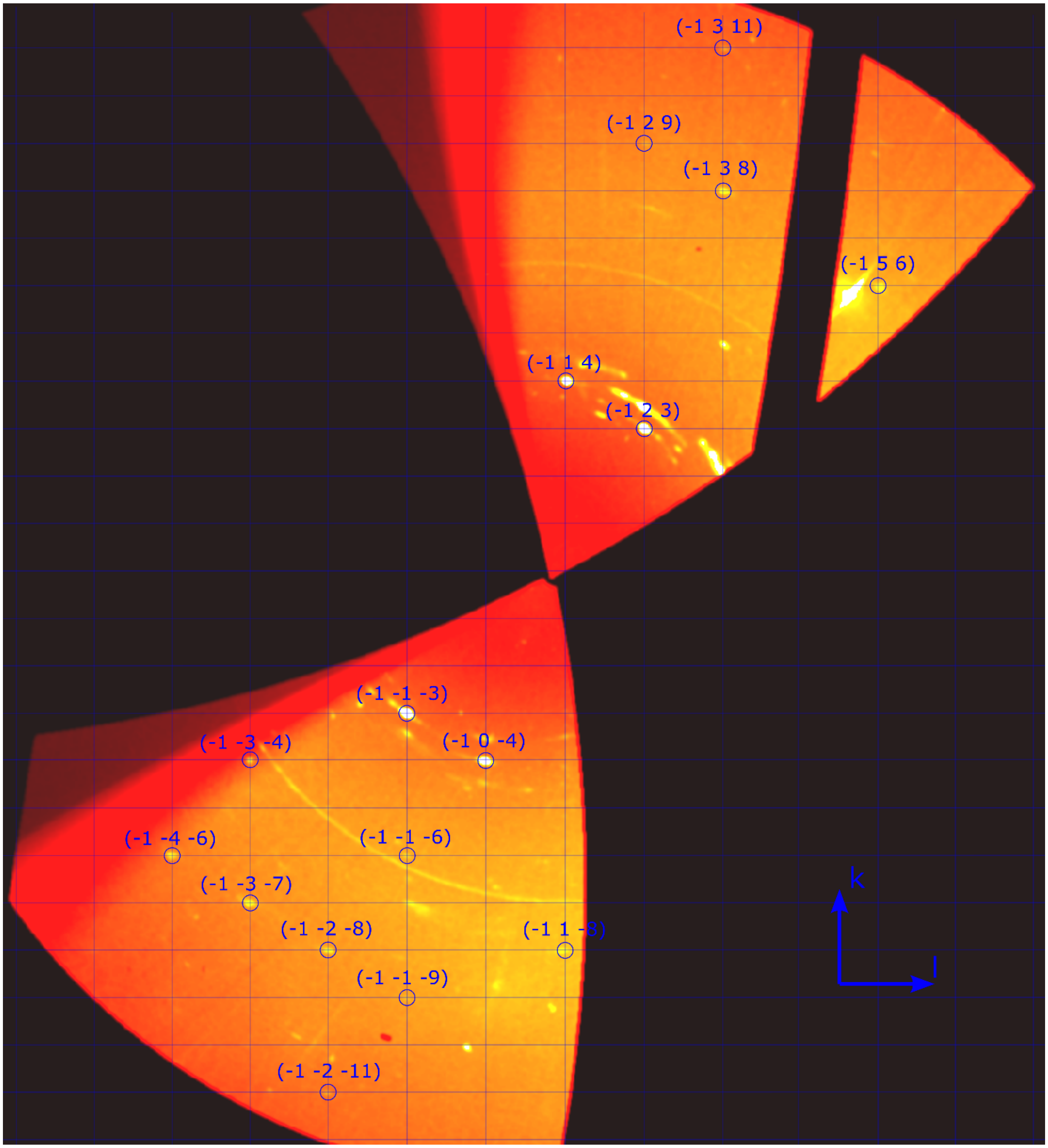

Supplementary Figure 1: Slice of the (-1kl) reciprocal space of an ε-N_2 single-crystal at 54 GPa.

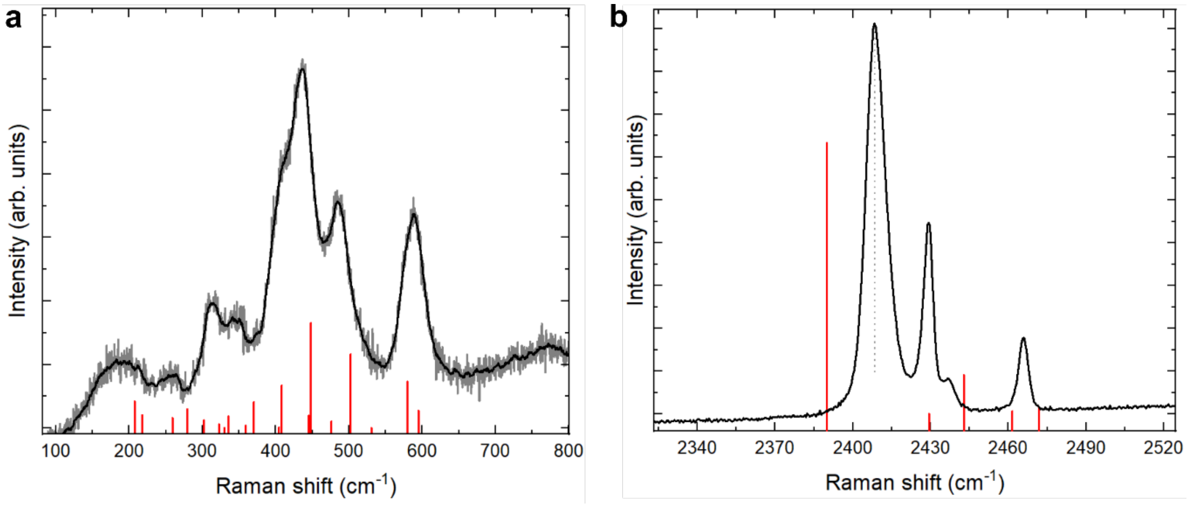

Supplementary Figure 2: Raman spectra collected on ζ-N_2 at 70 GPa. These cover the low (**a**) and high frequency (**b**) regions. In **a**, the raw data is shown in light grey, while the black line shows a smoothing of the raw data. In b), the dotted line at a frequency of 2408 cm^{-1} serves as a guide to the eyes to better see the asymmetric form of the peak. The red vertical lines are the positions of the 24 Raman modes of ζ-N_2 at 70 GPa calculated from DFT. The calculated frequencies are known to systematically underestimate the experimental frequencies by 2-3%[2]. To simplify the comparison, the calculated frequencies were shifted by 2.3%. The intensity scaling for the calculated modes was done independently for **a** and **b**. The calculated modes are in good qualitative agreement with the experimental data, both regarding their Raman shift and their intensity. Source data are provided as a Source Data file.

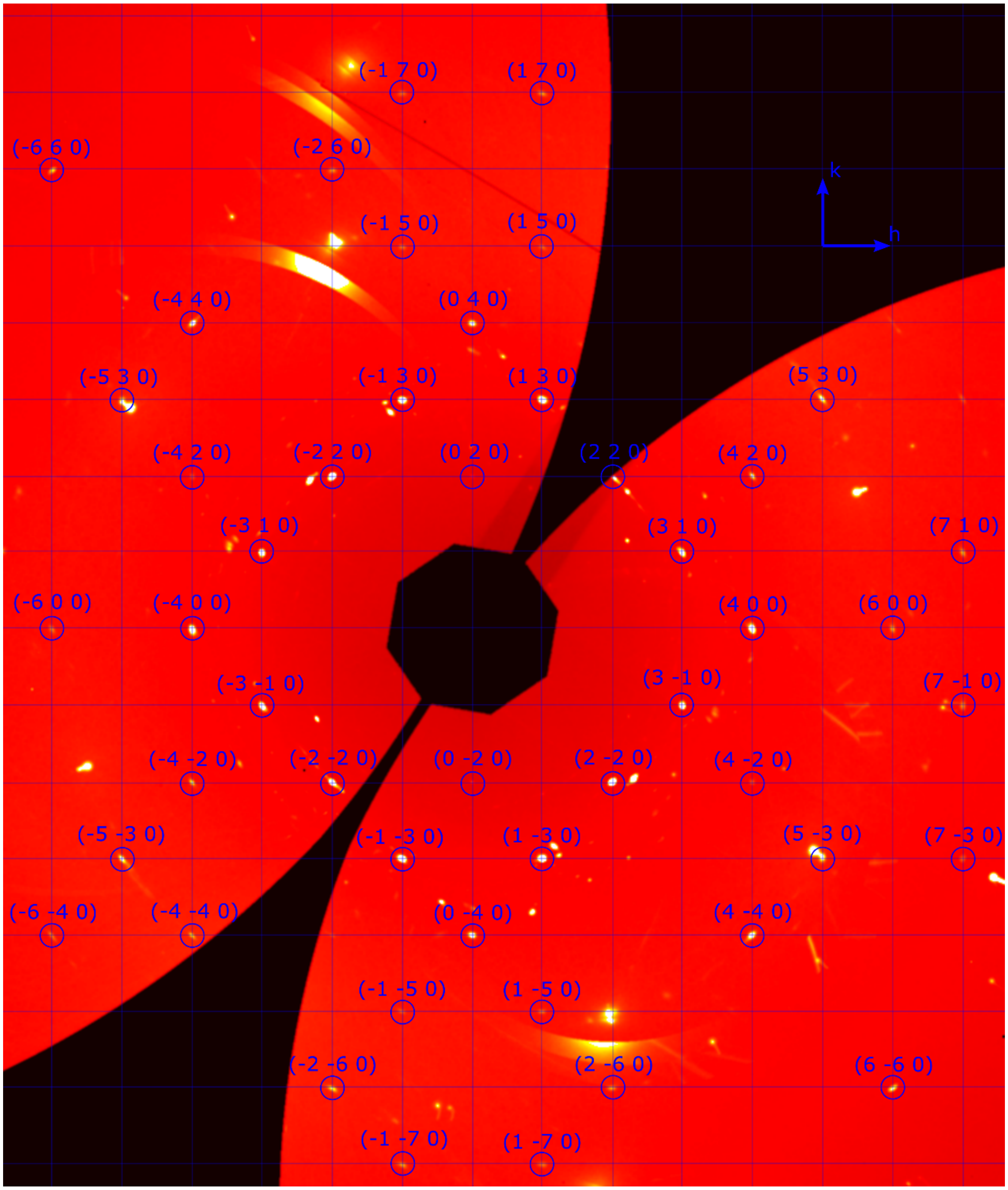

Supplementary Figure 3: Slice of the (hk0) reciprocal space of a ζ-N_2 single-crystal at 63 GPa. In agreement with the reflections conditions of the *C*2/*c* space group, reflections are only observed for h + k = 2n.

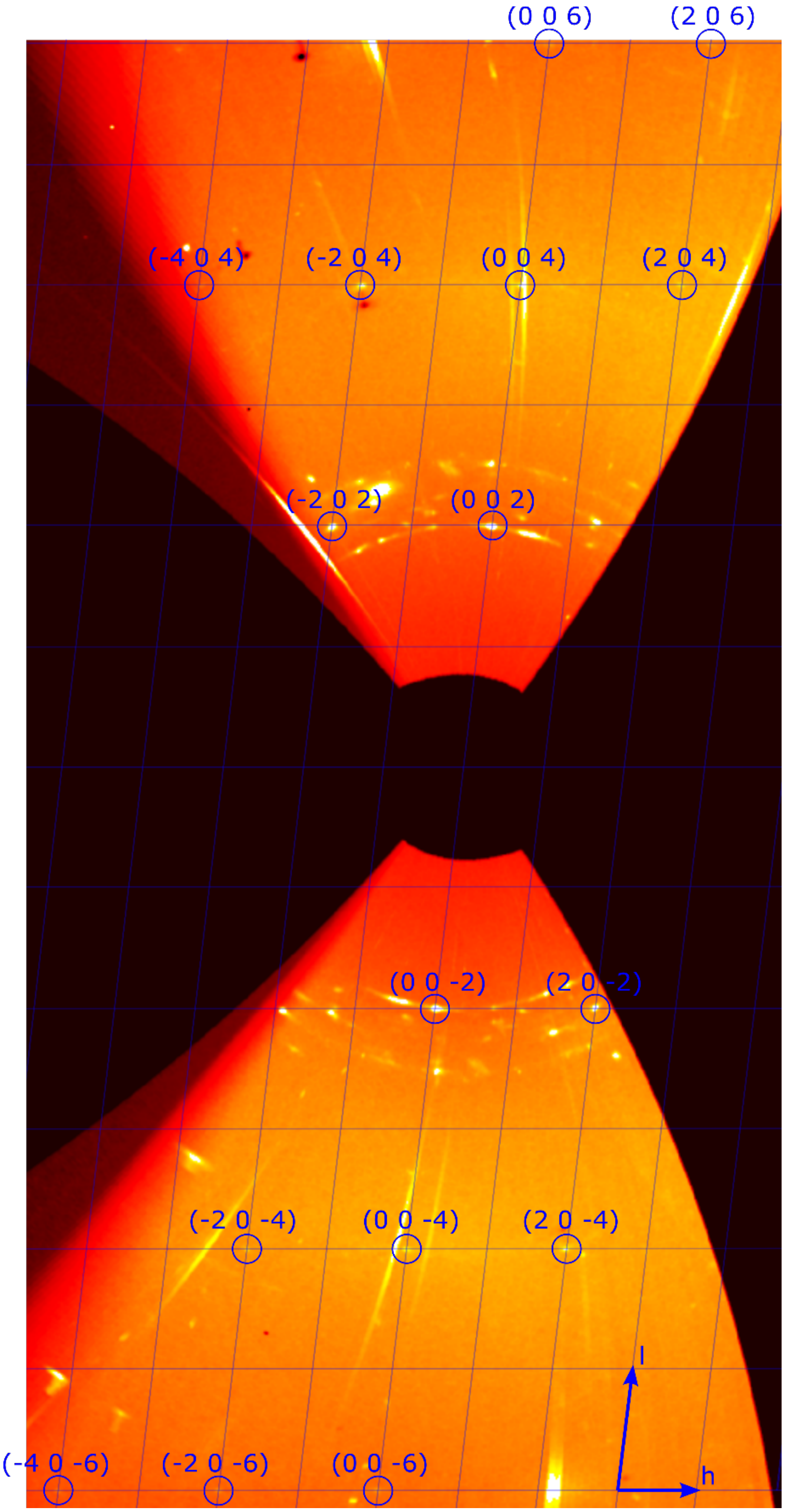

Supplementary Figure 4: Slice of the (h0l) reciprocal space of a ζ-N_2 single-crystal at 70 GPa. In agreement with the reflections conditions of the *C*2/*c* space group, reflections are only observed for h + k = 2n and h,l = 2n.

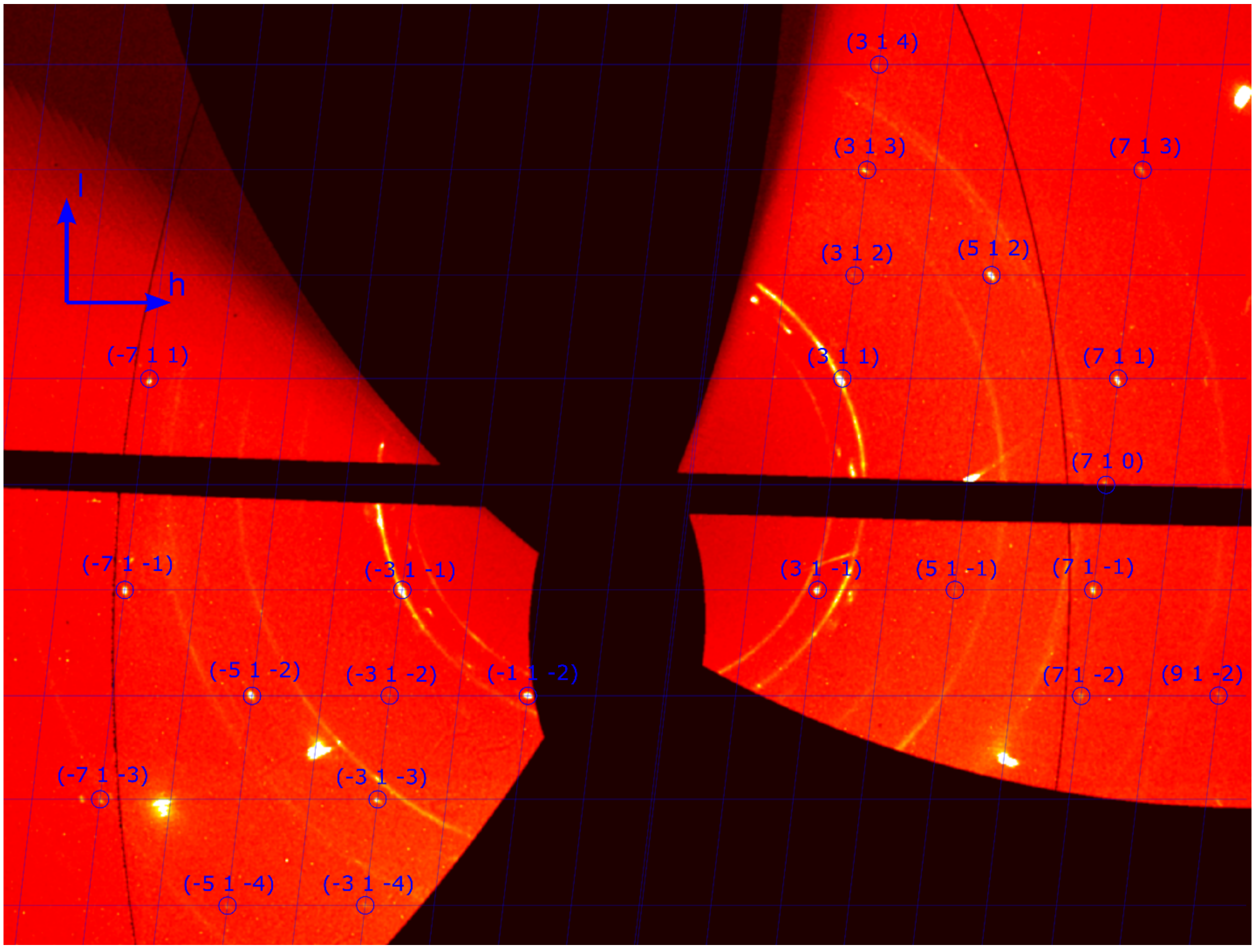

Supplementary Figure 5: Slice of the (hk0) reciprocal space of a ζ-N_2 single-crystal at 86 GPa. In agreement with the reflections conditions of the *C*2/*c* space group, reflections are only observed for h + k = 2n.

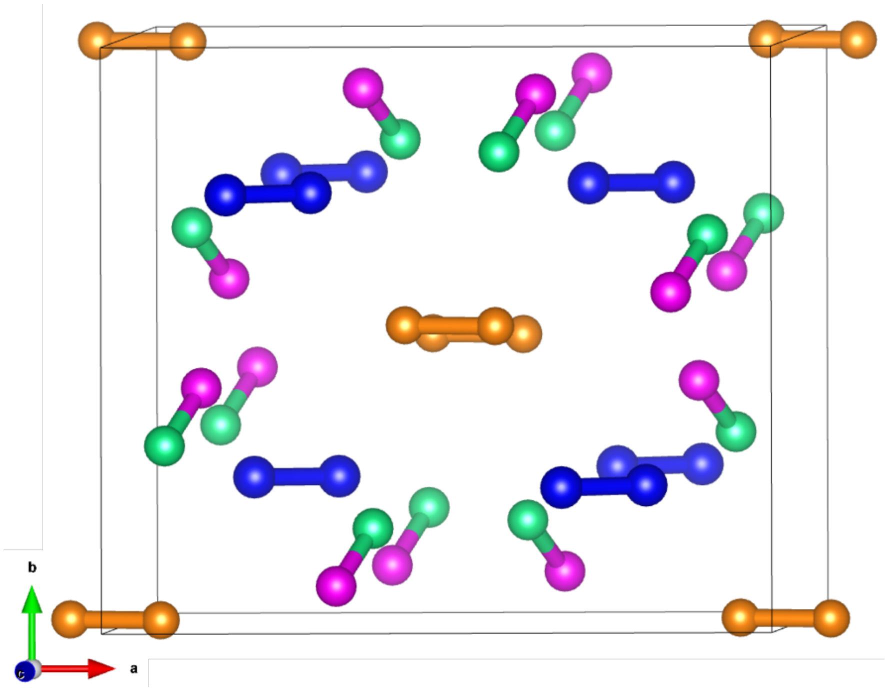

Supplementary Figure 6: Crystal structure of ζ-N_2 at 63 GPa with the atoms drawn with 50% probability ellipsoids. The blue, pink, orange and green spheres correspond to the N1, N2, N3 and N4 atoms, respectively. The corresponding crystallographic data has been submitted under the deposition number CCDC 2237807.

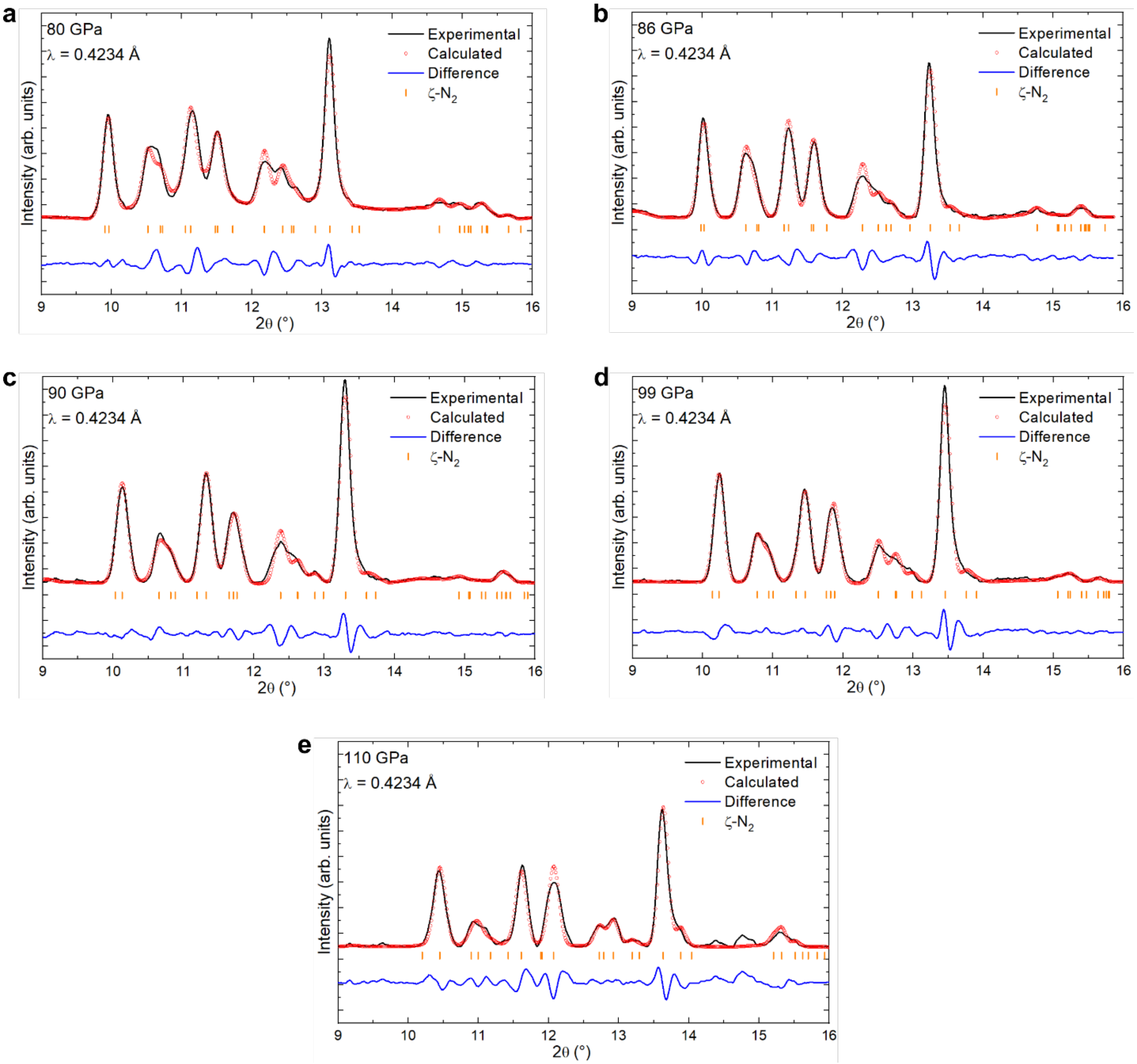

Supplementary Figure 7: Le Bail analysis of the powder X-ray diffraction data originating from Gregoryanz *et al*.[3] The refinement of the monoclinic unit cell parameters of ζ-N_2 was made for pressures of **a** 80 GPa, **b** 86 GPa, **c** 90 GPa, **d** 99 GPa and **e** 110 GPa. Source data are provided as a Source Data file.

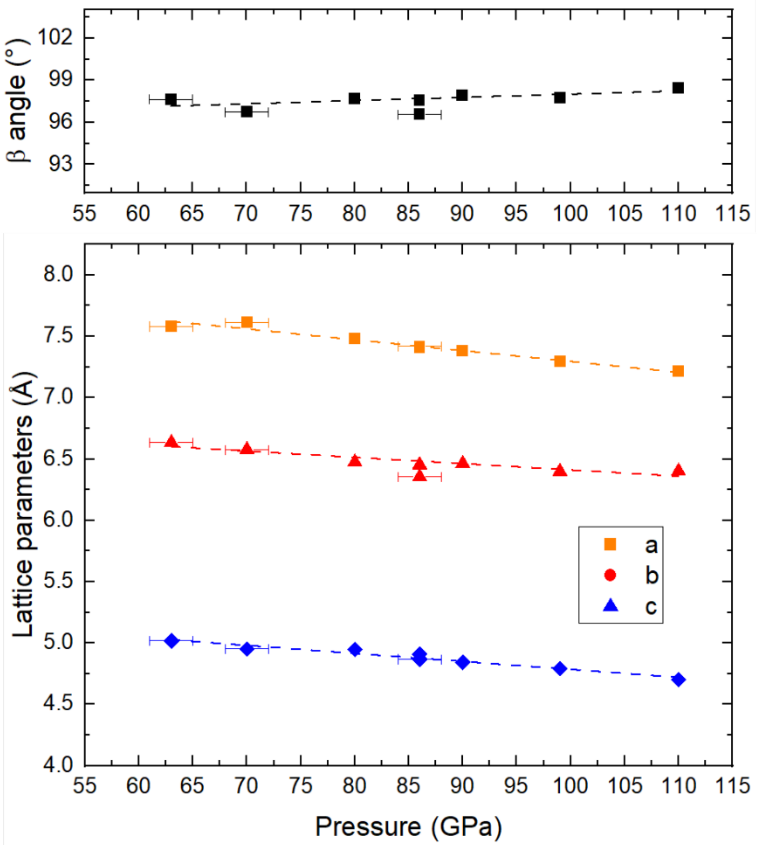

Supplementary Figure 8: Evolution of the experimentally-determined lattice parameters of ζ-N_2 with pressure. The *y*-axis error bars are smaller than the size of the points. The pressure uncertainty for the datapoints between 80 and 110 GPa, which were derived from the raw data presented in Gregoryanz *et al*., was not reported. The pressure uncertainty on the datapoints obtained from single-crystal X-ray diffraction is ±2 GPa. Source data are provided as a Source Data file.

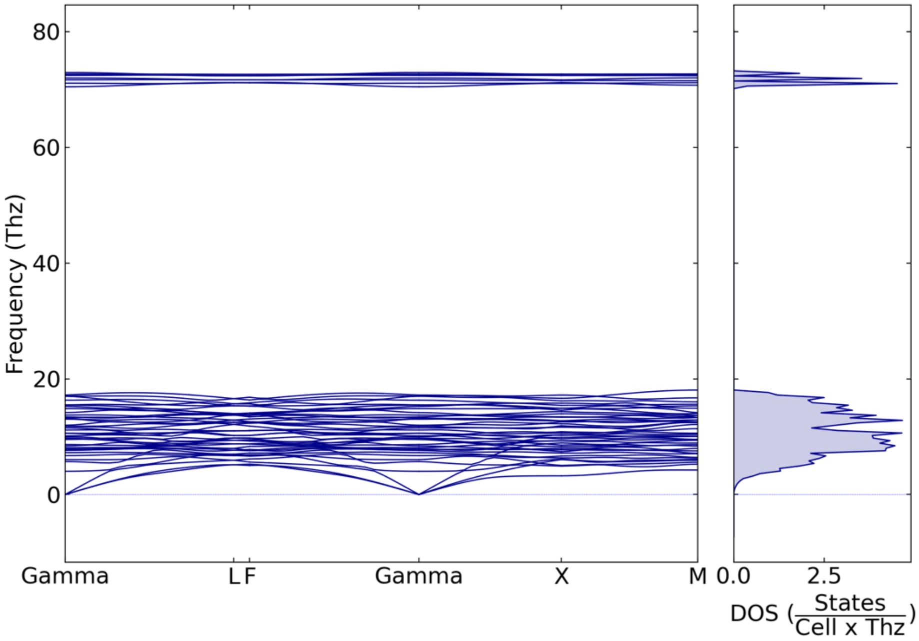

Supplementary Figure 9: Phonon dispersion relation for ζ-N_2 at 70 GPa, calculated with Phonopy[4]. Source data are provided as a Source Data file.

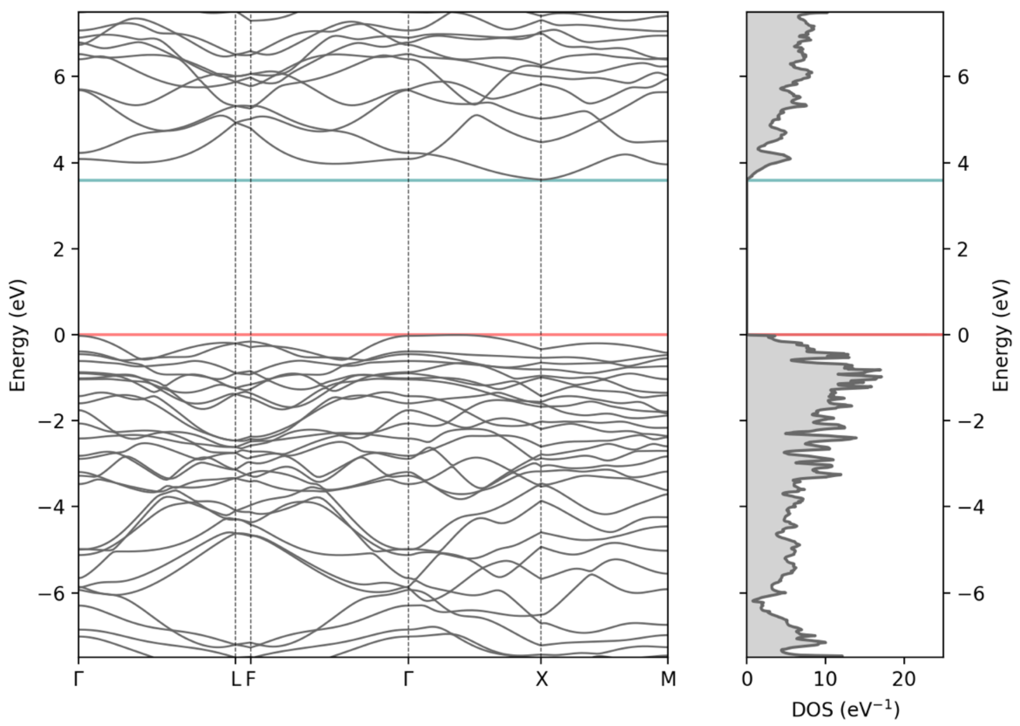

Supplementary Figure 10: Electronic band structure and density of states of ζ-N_2 at 70 GPa, calculated using PBE potential. Based on these calculations, ζ-N_2 would have an indirect band gap of 3.6 eV (HSE: 5.3 eV) at this pressure. More accurate calculations using the HSE functional predict band gap of ζ-N_2 to be 5.3 eV, as specified in the main text. Source data are provided as a Source Data file.

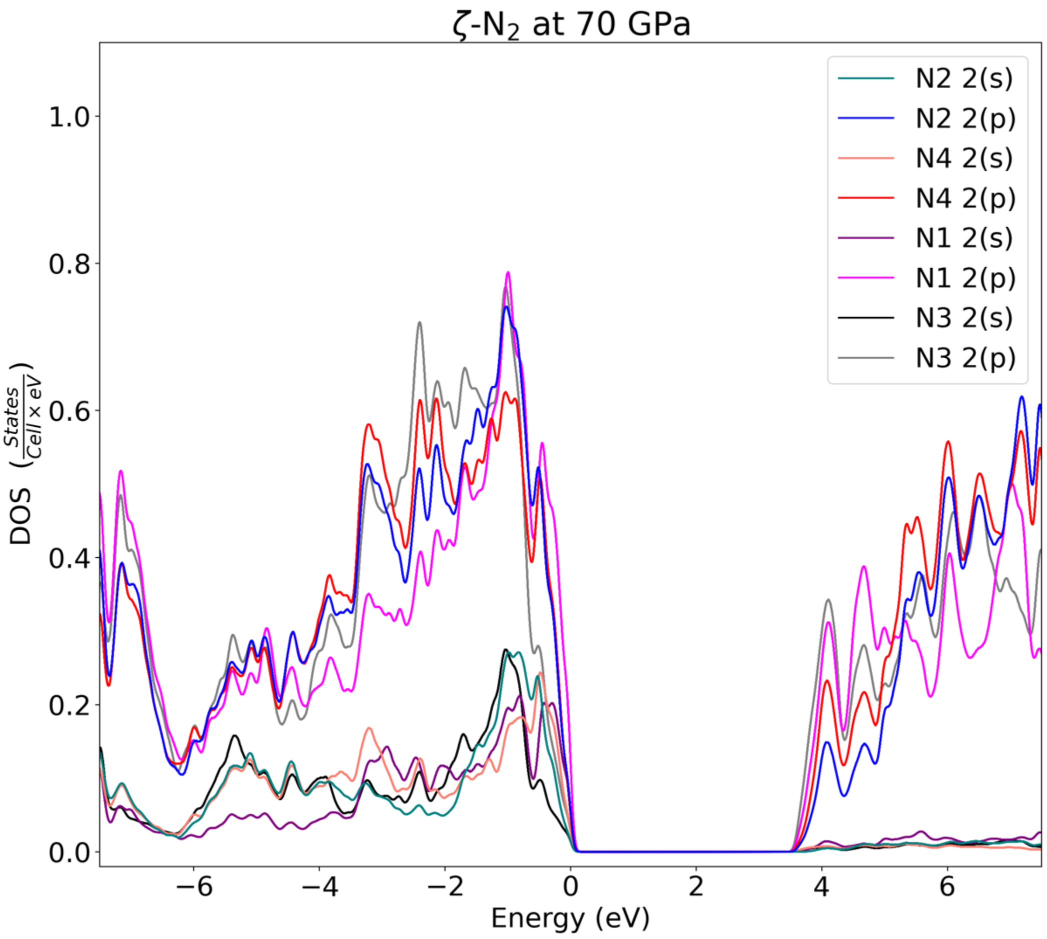

Supplementary Figure 11: Orbital project electronic density of states (pDOS) for ζ-N_2 at 70 GPa. A smearing of 0.005 Ry is used in the calculation of the pDOS. Source data are provided as a Source Data file.

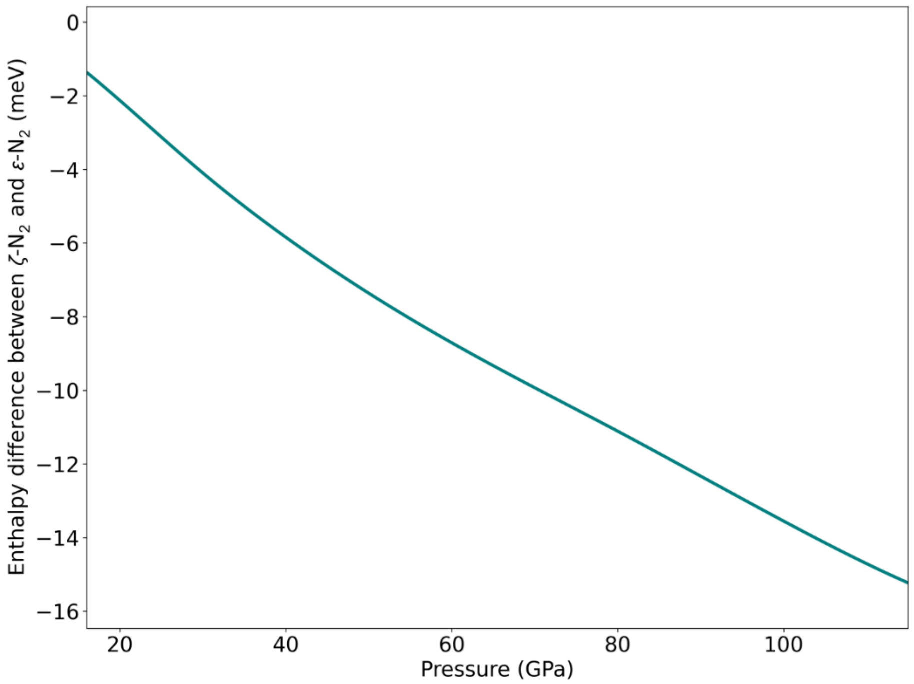

Supplementary Figure 12: Calculated static enthalpy difference between ζ- and ε-N_2. It is found that ζ-N_2 is marginally more stable than ε-N_2 across the whole calculated pressure range. The static calculations are in qualitative agreement with experimental measurements at low temperature which suggest the transition from ε-N_2 to ζ-N_2 to occur at 18.27 GPa at 0 K[5]. Source data are provided as a Source Data file.

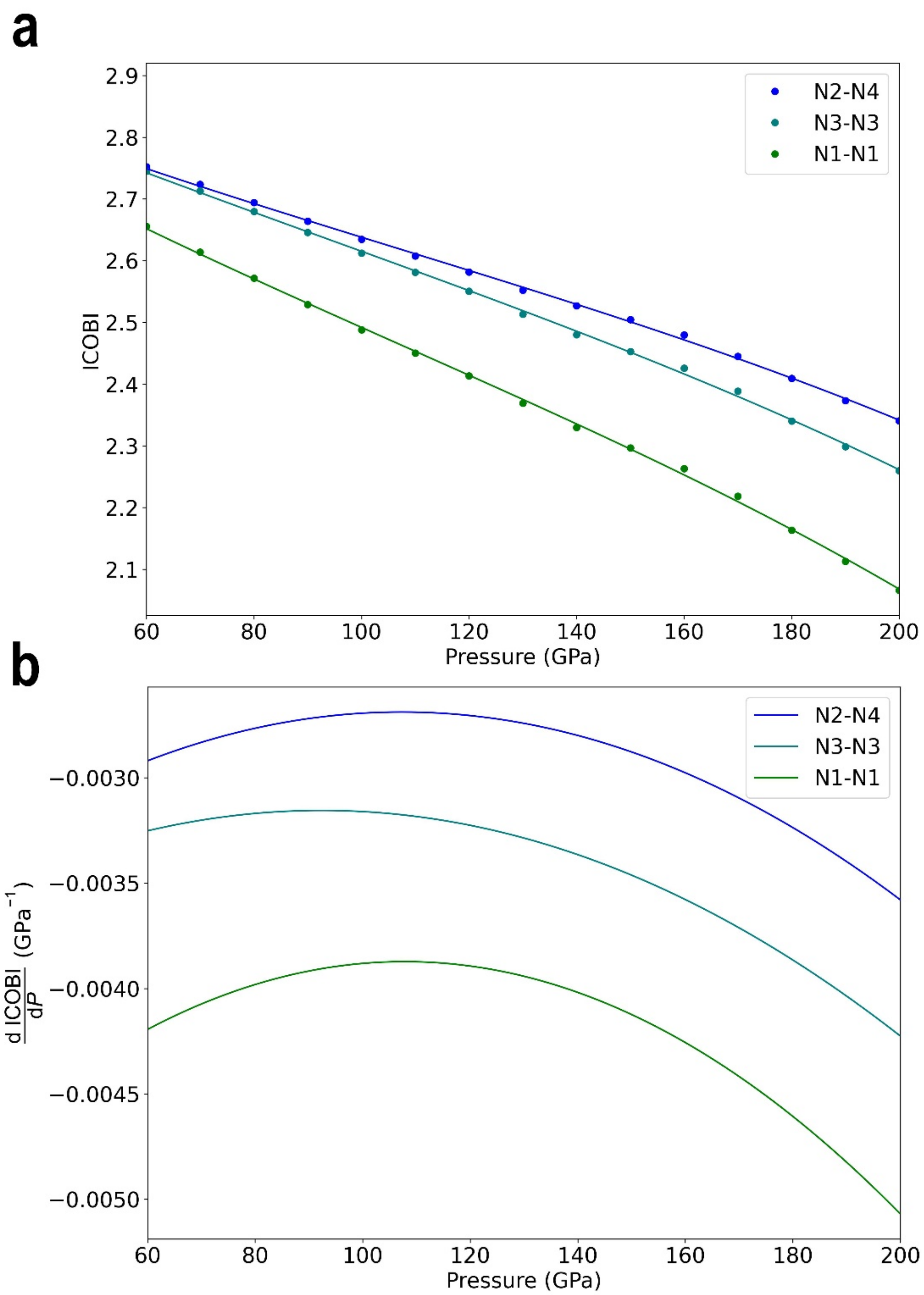

Supplementary Figure 13: Calculated integrated crystal orbital bond index (ICOBI) and its derivative with respect to pressure. **a** ICOBI values of the three crystallographically distinct N_2 molecules in ζ-N_2 between 60 to 200 GPa. The datapoints are fitted with a spline (full line). **b** The derivative of the N_2 molecules' ICOBI spline function with respect to pressure. For all three molecules, an acceleration of the decrease of ICOBI value is seen at pressures from 90 and 110 GPa. Source data are provided as a Source Data file.

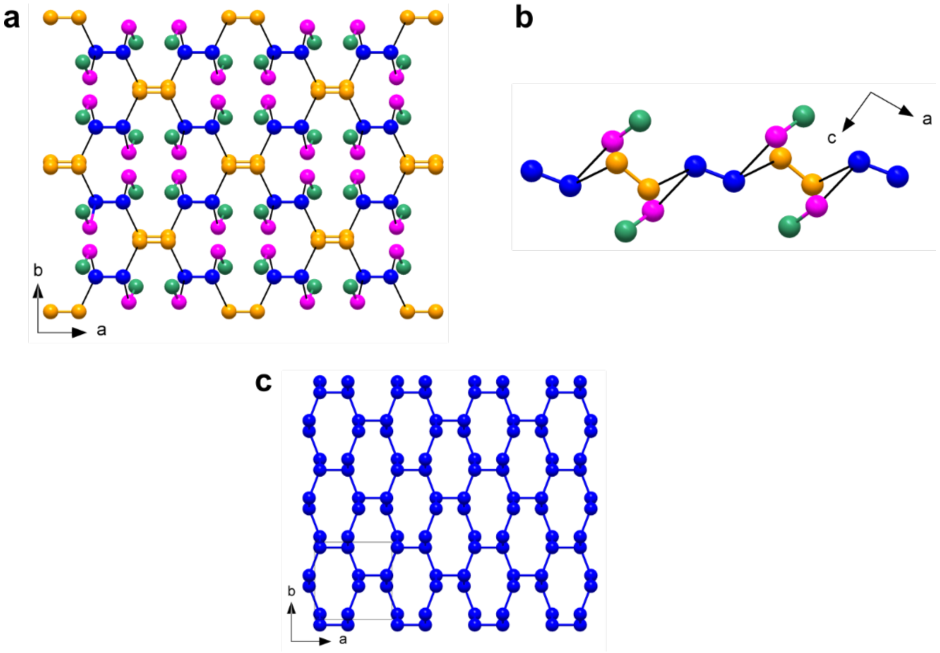

Supplementary Figure 14: Crystal structure similarities between ζ-N_2 and cg-N. **a** Calculated structure of ζ-N_2 at 150 GPa, with the blue, pink, green and orange spheres corresponding to the N1, N2, N3 and N4 atoms, viewed along the *c*-axis. The black lines represent the N_2 molecules identified by ELF to be linked through an isosurface value equal or greater than 0.5. These are forming infinite 1D strands, as shown in **b**. These strands are running parallel to the [-13 0 6] direction. **c** The crystal structure of cg-N at 115 GPa. Similarities can be seen between cg-N and ζ-N_2 when the latter is viewed along the *c*-axis.

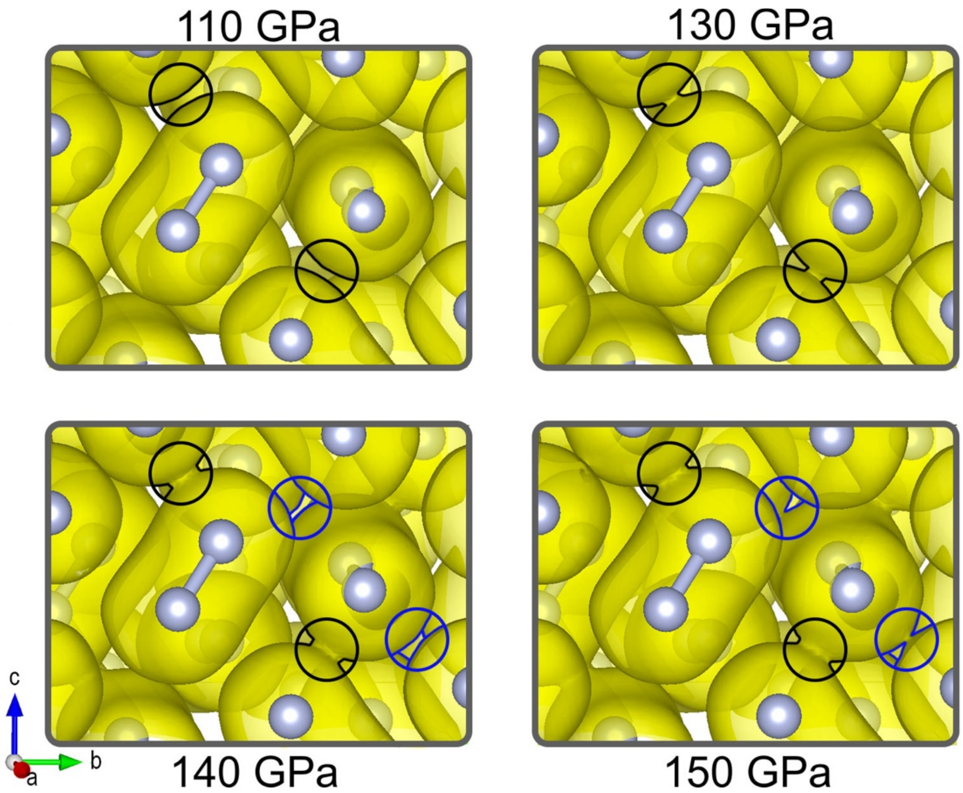

Supplementary Figure 15: Electron localization function (ELF) isosurfaces of 0.5 plotted at pressures of 110, 130, 140 and 150 GPa. Black and blue circles were added to highlight the regions where N_2 molecules' ELF isosurfaces start to connect at 130 GPa and at 150 GPa, respectively. Source data are provided as a Source Data file.

Supplementary Discussion

A- and B-level alerts were found by CheckCif upon verifying the submitted CIFs. These alerts, listed below and explained, are typical for data collected in diamond anvil cells.

ε-N_2 at 54 GPa (CCDC 2280044)

start Validation Reply Form
_vrf_ATOM007_I
;
PROBLEM: _atom_site_aniso_label is missing
RESPONSE: The ratio of reflections over parameters does not allow to refine
the thermal parameters of the nitrogen atoms anisotropically. This is
because this measurement was performed at high pressure which, due to the high
pressure apparatus, limits the theta range. Indeed, the diamond anvil cell
metallic body typically shadows more than 60% of the reflections.
;
_vrf_PLAT029_I
;
PROBLEM: _diffrn_measured_fraction_theta_full value Low .   0.760 Why?
RESPONSE: This measurement was performed at high pressure which, due to the high
pressure apparatus, limits the theta range. Indeed, the diamond anvil cell
metallic body typically shadows more than 60% of the reflections.
;
end Validation Reply Form

ζ-N_2 at 63 GPa (CCDC 2237807)

start Validation Reply Form
_vrf_ATOM007_I
;
PROBLEM: _atom_site_aniso_label is missing
RESPONSE: The ratio of reflections over parameters does not allow to refine
the thermal parameters of the nitrogen atoms anisotropically. This is
because this measurement was performed at high pressure which, due to the high
pressure apparatus, limits the theta range. Indeed, the diamond anvil cell
metallic body typically shadows more than 60% of the reflections.
;
_vrf_PLAT029_I
;
PROBLEM: _diffrn_measured_fraction_theta_full value Low .   0.590 Why?
RESPONSE: This measurement was performed at high pressure which, due to the high

pressure apparatus, limits the theta range. Indeed, the diamond anvil cell
metallic body typically shadows more than 60% of the reflections.
;
end Validation Reply Form

ζ-N_2 at 70 GPa (CCDC 2237808)
start Validation Reply Form
_vrf_ATOM007_I
;
PROBLEM: _atom_site_aniso_label is missing
RESPONSE: The ratio of reflections over parameters does not allow to refine
the thermal parameters of the nitrogen atoms anisotropically. This is
because this measurement was performed at high pressure which, due to the high
pressure apparatus, limits the theta range. Indeed, the diamond anvil cell
metallic body typically shadows more than 60% of the reflections.
;
_vrf_PLAT029_I
;
PROBLEM: _diffrn_measured_fraction_theta_full value Low . 0.450 Why?
RESPONSE: This measurement was performed at high pressure which, due to the high
pressure apparatus, limits the theta range. Indeed, the diamond anvil cell
metallic body typically shadows more than 60% of the reflections.
;
_vrf_PLAT088_I
;
PROBLEM: Poor Data / Parameter Ratio .... 6.41 Note
RESPONSE: This measurement was performed at high pressure which, due to the high
pressure apparatus, limits the theta range. Indeed, the diamond anvil cell
metallic body typically shadows more than 60% of the reflections.
;
end Validation Reply Form

ζ-N_2 at 86 GPa (CCDC 2261391)
start Validation Reply Form
_vrf_ATOM007_I
;
PROBLEM: _atom_site_aniso_label is missing
RESPONSE: The ratio of reflections over parameters does not allow to refine
the thermal parameters of the nitrogen atoms anisotropically. This is
because this measurement was performed at high pressure which, due to the high

pressure apparatus, limits the theta range. Indeed, the diamond anvil cell
metallic body typically shadows more than 60% of the reflections.
;
_vrf_PLAT029_I
;
PROBLEM: _diffrn_measured_fraction_theta_full value Low . 0.560 Why?
RESPONSE: This measurement was performed at high pressure which, due to the high
pressure apparatus, limits the theta range. Indeed, the diamond anvil cell
metallic body typically shadows more than 60% of the reflections.
;
_vrf_PLAT027_I
;
PROBLEM: _diffrn_reflns_theta_full value (too) Low ...... 7.23 Degree
RESPONSE: This measurement was performed at high pressure which, due to the high
pressure apparatus, limits the theta range. Indeed, the diamond anvil cell
metallic body typically shadows more than 60% of the reflections.
;
_vrf_PLAT088_I
;
PROBLEM: Poor Data / Parameter Ratio .... 6.76 Note
RESPONSE: This measurement was performed at high pressure which, due to the high
pressure apparatus, limits the theta range. Indeed, the diamond anvil cell
metallic body typically shadows more than 60% of the reflections.
;
end Validation Reply Form

Supplementary References

1. Mills, R. L., Olinger, B. & Cromer, D. T. Structures and phase diagrams of N2 and CO to 13 GPa by x-ray diffraction. *J. Chem. Phys.* **84**, 2837 (1986).

2. Lazzeri, M. & Mauri, F. First-Principles Calculation of Vibrational Raman Spectra in Large Systems: Signature of Small Rings in Crystalline SiO2. *Phys. Rev. Lett.* **90**, 036401 (2003).

3. Gregoryanz, E. *et al.* High P-T transformations of nitrogen to 170GPa. *J. Chem. Phys.* **126**, 184505 (2007).

4. Togo, A. & Tanaka, I. First principles phonon calculations in materials science. *Scr. Mater.* **108**, 1–5 (2015).

5. Bini, R., Ulivi, L., Kreutz, J. & Jodl, H. J. High-pressure phases of solid nitrogen by Raman and infrared spectroscopy. *J. Chem. Phys.* **112**, 8522 (2000).
