# Supplementary material for: Structure determination of ζ-N2 from single-crystal X-ray diffraction and theoretical suggestion for the formation of amorphous nitrogen
Source: Nat Commun. 2023 Oct 5;14:6207. doi: 10.1038/s41467-023-41968-2 (PMC10556017; doi:10.1038/s41467-023-41968-2)
Supplement: Supplementary file 3 — Description of Additional Supplementary Files Document [file 41467_2023_41968_MOESM3_ESM.pdf]

Description of Additional Supplementary Files

File name: Supplementary Data 1

Description: Crystallographic Information Files (CIFs) for ε-N_2 at 54 GPa as well as ζ-N_2 at 63, 70 and 86 GPa.

File name: Supplementary Data 2

Description: Electron Localization Functions (ELFs) of ζ-N_2 at 110, 130, 140 and 150 GPa.
