# Supplementary material for: Structure determination of ζ-N2 from single-crystal X-ray diffraction and theoretical suggestion for the formation of amorphous nitrogen
Source: Nat Commun. 2023 Oct 5;14:6207. doi: 10.1038/s41467-023-41968-2 (PMC10556017; doi:10.1038/s41467-023-41968-2)

checkCIF/PLATON report

Structure factors have been supplied for datablock(s) I

THIS REPORT IS FOR GUIDANCE ONLY. IF USED AS PART OF A REVIEW PROCEDURE FOR PUBLICATION, IT SHOULD NOT REPLACE THE EXPERTISE OF AN EXPERIENCED CRYSTALLOGRAPHIC REFEREE.

No syntax errors found.   CIF dictionary   Interpreting this report

Datablock: I

```
Bond precision:   N- N = 0.0063 A          Wavelength=0.28460

Cell:               a=7.417(5)      b=6.354(6)          c=4.869(2)
                    alpha=90        beta=96.57(4)       gamma=90
Temperature:        293 K

                 Calculated                     Reported
Volume           228.0(3)                       228.0(3)
Space group      C 2/c                          C 1 2/c 1
Hall group       -C 2yc                         -C 2yc
Moiety formula   N2                             ?
Sum formula      N2                             N2
Mr               28.02                          28.00
Dx,g cm-3        3.265                          3.265
Z                16                             16
Mu (mm-1)        0.072                          0.070
F000             224.0                          224.0
F000'            223.79
h,k,lmax         9,7,6                          9,6,5
Nref             230                            115
Tmin,Tmax                                       0.904,1.000
Tmin'

Correction method= # Reported T Limits: Tmin=0.904 Tmax=1.000
AbsCorr = MULTI-SCAN

Data completeness= 0.500           Theta(max)= 10.240

                                                     wR2(reflections)=
R(reflections)= 0.0616( 77)                          wR= 0.0579( 115)

S = 2.490                  Npar= 17
```

The following ALERTS were generated. Each ALERT has the format
test-name_ALERT_alert-type_alert-level.
Click on the hyperlinks for more details of the test.

Alert level A

ATOM007_ALERT_1_A _atom_site_aniso_label is missing
Unique label identifying the atom site.

Author Response: The ratio of reflections over parameters does not allow to refine the thermal parameters of the nitrogen atoms anisotropically. This is because this measurement was performed at high pressure which, due to the high pressure apparatus, limits the theta range. Indeed, the diamond anvil cell metallic body typically shadows more than 60% of the reflections.

PLAT027_ALERT_3_A _diffrn_reflns_theta_full value (too) Low ...... 7.23 Degree

Author Response: This measurement was performed at high pressure which, due to the high pressure apparatus, limits the theta range. Indeed, the diamond anvil cell metallic body typically shadows more than 60% of the reflections.

PLAT029_ALERT_3_A _diffrn_measured_fraction_theta_full value Low . 0.560 Why?

Author Response: This measurement was performed at high pressure which, due to the high pressure apparatus, limits the theta range. Indeed, the diamond anvil cell metallic body typically shadows more than 60% of the reflections.

Alert level B

PLAT088_ALERT_3_B Poor Data / Parameter Ratio .... 6.76 Note
N1 N2 N4 N3

Author Response: This measurement was performed at high pressure which, due to the high pressure apparatus, limits the theta range. Indeed, the diamond anvil cell metallic body typically shadows more than 60% of the reflections.

Alert level C

GOODF01_ALERT_2_C The least squares goodness of fit parameter lies
outside the range 0.80 <> 2.00
Goodness of fit given = 2.490
PLAT053_ALERT_1_C Minimum Crystal Dimension Missing (or Error) ... Please Check
PLAT054_ALERT_1_C Medium Crystal Dimension Missing (or Error) ... Please Check
PLAT055_ALERT_1_C Maximum Crystal Dimension Missing (or Error) ... Please Check
PLAT799_ALERT_4_C Numeric Label on Displacement Par. Record ...... ? Check

Alert level G

```
ABSMU01_ALERT_1_G  Calculation of _exptl_absorpt_correction_mu
                not performed for this radiation type.
PLAT005_ALERT_5_G No Embedded Refinement Details Found  in the CIF     Please Do !
PLAT092_ALERT_4_G Check: Wavelength Given is not Cu,Ga,Mo,Ag,In Ka    0.28460 Ang.
PLAT199_ALERT_1_G Reported _cell_measurement_temperature ..... (K)        293 Check
PLAT200_ALERT_1_G Reported   _diffrn_ambient_temperature ..... (K)        293 Check
PLAT808_ALERT_5_G No Parseable SHELXL Style Weighting Scheme Found     Please Check
PLAT883_ALERT_1_G No Info/Value for _atom_sites_solution_primary .     Please Do !
PLAT911_ALERT_3_G Missing FCF Refl Between Thmin & STh/L=    0.600         96 Report
PLAT912_ALERT_4_G Missing # of FCF Reflections Above STh/L=  0.600          6 Note
PLAT913_ALERT_3_G Missing # of Very Strong Reflections in FCF ....          2 Note
PLAT929_ALERT_5_G No Weight Pars,Obs and Calc R1,wR2,S not Checked          ! Info
PLAT966_ALERT_5_G Note: Non-Standard (i.e. 2.0) OMIT Threshold  of        3.0 Sig(I)
```

```
   3 ALERT level A = Most likely a serious problem - resolve or explain
   1 ALERT level B = A potentially serious problem, consider carefully
   5 ALERT level C = Check. Ensure it is not caused by an omission or oversight
  12 ALERT level G = General information/check it is not something unexpected

   8 ALERT type 1 CIF construction/syntax error, inconsistent or missing data
   1 ALERT type 2 Indicator that the structure model may be wrong or deficient
   5 ALERT type 3 Indicator that the structure quality may be low
   3 ALERT type 4 Improvement, methodology, query or suggestion
   4 ALERT type 5 Informative message, check
```

It is advisable to attempt to resolve as many as possible of the alerts in all categories. Often the minor alerts point to easily fixed oversights, errors and omissions in your CIF or refinement strategy, so attention to these fine details can be worthwhile. In order to resolve some of the more serious problems it may be necessary to carry out additional measurements or structure refinements. However, the purpose of your study may justify the reported deviations and the more serious of these should normally be commented upon in the discussion or experimental section of a paper or in the "special_details" fields of the CIF. checkCIF was carefully designed to identify outliers and unusual parameters, but every test has its limitations and alerts that are not important in a particular case may appear. Conversely, the absence of alerts does not guarantee there are no aspects of the results needing attention. It is up to the individual to critically assess their own results and, if necessary, seek expert advice.

Publication of your CIF in IUCr journals

A basic structural check has been run on your CIF. These basic checks will be run on all CIFs submitted for publication in IUCr journals (*Acta Crystallographica*, *Journal of Applied Crystallography*, *Journal of Synchrotron Radiation*); however, if you intend to submit to *Acta Crystallographica Section C* or *E* or *IUCrData*, you should make sure that full publication checks are run on the final version of your CIF prior to submission.

Publication of your CIF in other journals

Please refer to the *Notes for Authors* of the relevant journal for any special instructions relating to CIF submission.

PLATON version of 28/11/2022; check.def file version of 28/11/2022

Datablock I - ellipsoid plot

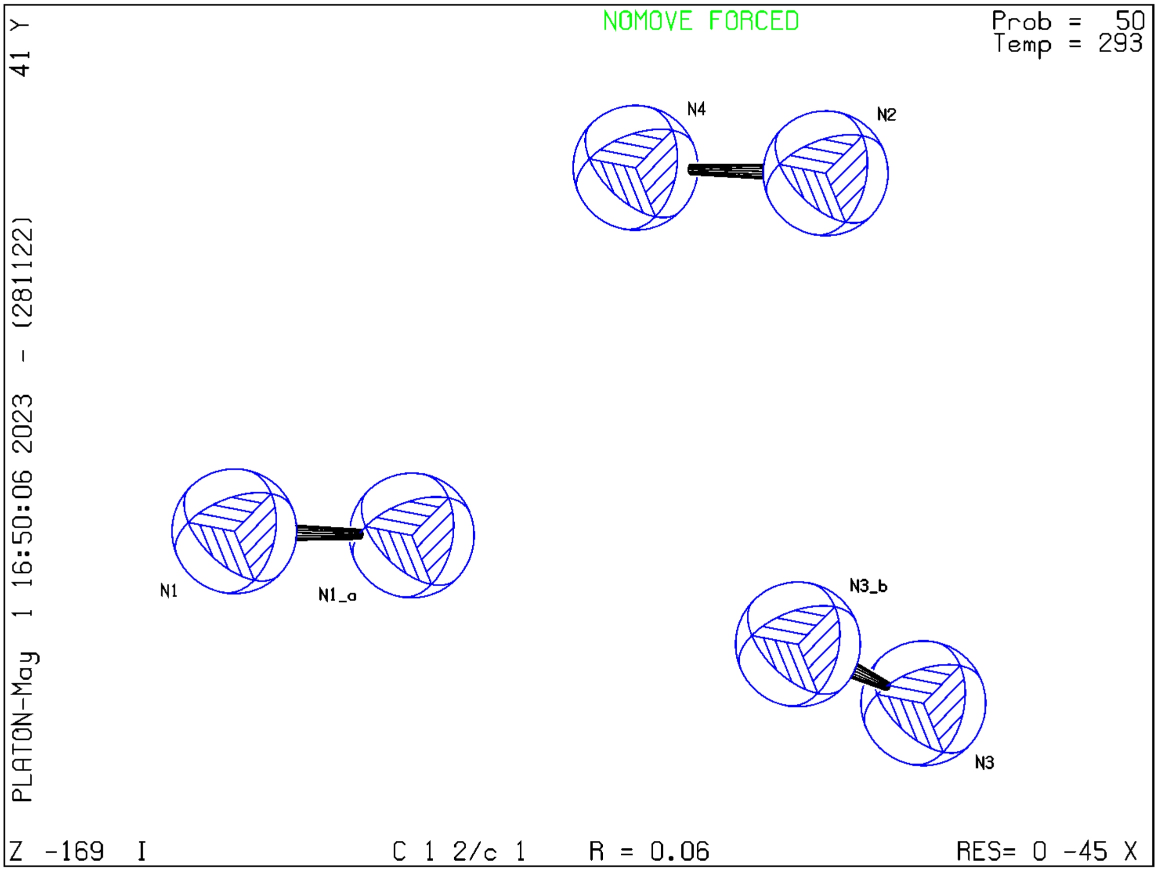

Supplement: Supplementary file 4 — Supplementary Dataset 1 [file checkcif.pdf › cifs for submission/86 GPa/checkcif.pdf]
